# Barriers and facilitators to feeling safe for inpatients: a model based on a qualitative meta-synthesis

**DOI:** 10.3389/fpubh.2024.1308258

**Published:** 2024-02-28

**Authors:** Lupei Yan, Li Liu, Fang Wang, Fanyu Zhao, Xiuying Hu

**Affiliations:** Innovation Center of Nursing Research, Nursing Key Laboratory of Sichuan Province, West China Hospital, West China School of Nursing, Sichuan University, Chengdu, China

**Keywords:** feeling safe, inpatients, qualitative meta-synthesis, patient safety, model

## Abstract

**Objectives:**

To review and synthesize qualitative research exploring patients’ safe experience and construct a model to present barriers and facilitators to feeling safe for inpatients.

**Design:**

A qualitative met-synthesis.

**Methods:**

We conducted a systematic electronic search of articles published in English with no date limitation across five databases (Ovid MEDLINE, EMBASE, Web of Science, CINAIL via EBSCO, and PsyINFO) in May 2023. Qualitative research focused on the safe experiences of inpatients was considered. Systematic searches yielded 8,132 studies, of which 16 articles were included. Two reviewers independently extracted and analyzed data. Qualitative meta-synthesis was performed through line-by-line coding of original texts, organizing codes into descriptive themes, and generating analytical themes.

**Results:**

We identified four themes and 11 sub-themes. Across the four themes, control included a barrier (Uncertainty) and two facilitators (Patient participation and safe care); responsible included three facilitators (Confidence in the profession, care for, and responsive); dignity included two barriers (Privacy and Neglect); stability included a barrier (Potential risk), and two facilitators (Harmonious and safe culture). We constructed a model to present the logical connection between these themes and related barriers and facilitators.

**Conclusion:**

Feeling safe for inpatients is a complex perception, including four themes: control, responsible, dignity, and stability. Surrounding four themes and related barriers and facilitators, we outline principles for creating a safe environment and present strategies for improving patients’ hospitalization experience and ensuring patient safety.

**Clinical relevance:**

This review provides valuable insight into the clinical practice and health policy and helps medical staff to identify and overcome the potential barriers to implementing interventions in safe care. In addition, the model comprehensively describes the nature and dimensions of feeling safe, informing high-quality care service and related research.

**Systematic review registration:**

Identifier, CRD42023435489.

## 1 Introduction

Patient safety is a hot public health topic and the core indicator of the quality of medical institutions (1). Keeping patients free from accidental or preventable harms caused by medical staff or other machines is a vital responsibility of personnel and hospitals. Previous studies mostly viewed patient safety from the perspective of healthcare professionals or hospitals, focusing on the report of adverse events, the incidence of medical errors, patient safety competency, and patient safety culture (1–3). However, healthcare professionals devoted themselves to improving the safety and quality of care and ignored what it meant to the patient. Patient safety may be much different from feeling safe for inpatients. From patients’ perspectives, they did not systematically learn medical knowledge and only knew what they felt (4).

In 1999, feeling safe was first described as a sense in which patients experienced no risk of physical or emotional harm (5). Subsequently, a concept analysis study defined feeling safe as an emotional state in which care awareness contributed to perceptions of safety and freedom from harm (4). Moreover, four attributes of feeling safe were identified: presence, trust, knowledge, and cared for. Suffering, environment, and relationship were the antecedents, and control, relaxed or calm, and hope were the consequences (4). Clarifying the concept of feeling safe contributed to increasing medical staff awareness of patients’ safe perceptions. Accordingly, interest in feeling safe has increased in the past years, and many studies have begun to explore the experiences and understandings of feeling safe for inpatients.

A qualitative study comprising five focus group discussions investigated 35 inpatients, identifying patients’ safe experiences across four core themes: (a) Patients who want to take the initiative in controlling their reception of information; (b) Healthcare providers who make the patient feel safe; (c) Hospital’s unstinted and generous support; and (d) Public sentiment about national healthcare and safety (6). This study found that patients felt safe not only because of the attitudes and professionalism of the medical staff but also the procedure, system, and support of the healthcare organizations (6). Another grounded theory study found that patients’ perception of safety arose from various care experiences involving specific actors: the patients and their caregivers, medical staff, and healthcare institutions (7). These care experiences and the quality of interaction between inpatients and related stakeholders were significant in forming patients’ safe perceptions (7).

Three qualitative descriptive articles showed that patients’ safe experiences were related to patients’ personhood, the working ways of nurses, and the hospital environment (8–10). During the COVID-19 Pandemic, a semi-structured interview described safety concerns experienced by inpatients and factors and outcomes of decisions about voicing safe concerns (11). This study reported the significance of open safety communication and credible response to patients and their caregivers who voiced concerns, indicating that factors influencing feeling safe involved staff characteristics, communication and coordination, and safe care expectations (11). Both these studies presented that feeling safe was a complex sense and related to many domains, but studies focused on different topics had different results.

Existing research about feeling safe mainly focused on explaining the concept connotations, identifying factors, and exploring safe concerns and outcomes. To our knowledge, no review has classified the facilitators or barriers to feeling safe for patients during hospitalization. Qualitative research captures experience and perception and forms a straight descriptive summary of text connotations organized in a way that best fits the original data (11, 12). Qualitative meta-syntheses synthesize novel interpretations of results based on the analysis of each article. These interpretations could not be seen in any one study, but they are integration and inferences derived from taking all of the research as a whole (12, 13). At present, the various findings of feeling safe for inpatients may mean there is a knowledge gap about the best safe practice. Therefore, this study aimed to examine and synthesize qualitative studies on the safe feelings of inpatients and constructed a model to connect the barriers and facilitators to the implementation of improving inpatients’ safe experiences.

## 2 Methods

### 2.1 Study design

We conducted a systematic review and qualitative meta-synthesis (CRD42023435489) and reported our results following the Preferred Reporting Items for Systematic Reviews and Meta-Analyses (PRISMA) (14) and the Enhancing transparency in reporting the synthesis of qualitative research (ENTREQ) statement (15). We used the research method because the qualitative meta-synthesis can collect data across multiple contexts, stimulate new conceptual understandings, develop theoretical models, and provide evidence for designing, evaluating, and implementing research programs (15). Ethical approval was not required for this meta-synthesis of existing qualitative articles.

### 2.2 Data sources

We conducted a systematic electronic search of articles published in English with no date limitation across five databases (Ovid MEDLINE, EMBASE, Web of Science, CINAIL via EBSCO, and PsyINFO) on May 21, 2023. The search strategy is shown in Supplementary Table 1. Briefly, the search included terms related to “hospital” and “feeling safe.” Additional potential qualitative studies were located by manually searching the reference lists of all included articles.

The eligibility criteria followed PICoS principles formulated by the Joanna Briggs Institute. The population (P) was inpatients or their caregivers (18 years and older). The interest of phenomena (I) was that inpatients felt safe. The context (Co) was the safe feelings related to the hospital. The study design (S) was qualitative studies. Qualitative studies included articles with grounded theory, ethnography, content or thematic analysis, phenomenology, hermeneutics, and primarily analyzing textual data (15). Articles only reporting healthcare professionals’ experiences and perspectives of safety in the hospital were excluded.

### 2.3 Study selection and data extraction

Search results were imported into Endnote X9 for the automatic removal of duplicates. Two reviewers independently screened titles and abstracts and performed full-text reviews to identify eligible articles. Then, results were pooled, and any discrepancies were resolved by discussion with a third reviewer. Eligible information was extracted into a spreadsheet which included: (a) general article characteristics; (b) study aims; (c) participants; (d) methods; and (e) themes and conclusions. The “themes and conclusions” text from the spreadsheet was imported into Nvivo 20 (QSR International).

### 2.4 Quality assessment

Two reviewers independently assessed eligible articles for methodological quality using the 10-item Critical Appraisal Skills Program (CASP) for qualitative studies checklist (16). Discrepancies were resolved by discussion and additional evaluation with a third reviewer. The CASP checklist provided a flexible tool to assess the credibility, rigor, and relevance of the heterogeneous studies (17). In line with existing qualitative meta-synthesis, a score of seven or higher on the CASP checklist was assessed as “reasonable quality (17).”

### 2.5 Data synthesis

We used Thomas and Hardens’ thematic synthesis method: (a) line-by-line coding of original texts; (b) organization of codes into descriptive themes; and (c) generation of analytical themes (18). First, original texts were repeatedly inspected in their original context and compared against the related text to ensure the accuracy of interpretation and the adequacy of the initial code. Accordingly, original codes were refined, and new codes were generated. Second, related codes were organized together to develop descriptive themes. Third, the consistency of descriptive themes in explaining patient safety experiences was examined. Existing codes in each theme were then classified and visually compared, and the analytic themes were generated and refined. In addition, we actively explored codes that did not fit the original contexts or themes and constructed new analytic themes as necessary. Finally, the positive and negative codes were identified to generate the barriers and facilitators to feeling safe for inpatients, and a model was further developed.

### 2.6 Rigor

The synthesis stage carefully followed Thomas and Hardens’ thematic synthesis method. The origin of the codes and quotes was tracked throughout the synthesis, coding was used, and illustrative quotes were shown. In addition, the researchers worked reflexively during the synthesis, discussing their biases and potential influence on the analysis.

## 3 Results

### 3.1 Study characteristics

**Figure 1:**
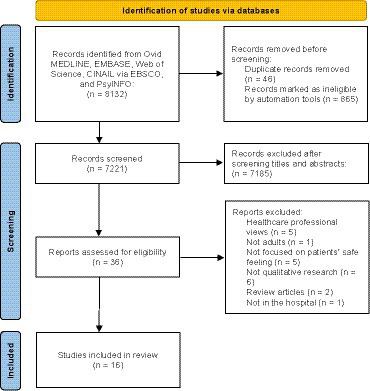
PRISMA flowchart of initial searches and inclusion. From Page et al. (19). For more information, visit: http://www.prisma-statement.org/.

**Figure 2:**
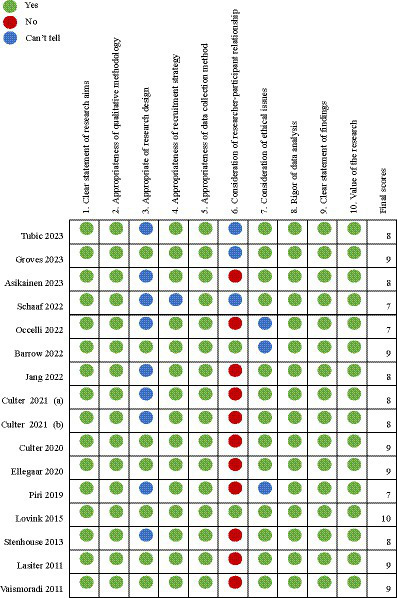
Results of quality assessment.

A total of 8,132 articles were identified through searching. After removing 911 duplicates and 7,205 studies that did not meet inclusion criteria, 16 were included for qualitative meta-synthesis. This process is shown in the Preferred Reporting Items for Systematic Reviews and Meta-Analyses (PRISMA) flowchart (Figure 1). The studies (2011–2023) were conducted in Europe (*n* = 9), Oceania (*n* = 3), North America (*n* = 2), and Asia (*n* = 2). In relation to the clinical settings, psychiatric wards (*n* = 7) and surgical wards (*n* = 3) were common. Various qualitative methodologies were used, and the most common method was interviews using thematic analysis (*n* = 7). The detailed study characteristics are shown in Table 1. The quality assessment showed that all studies met the “reasonable quality” criterion of the Critical Appraisal Skills Program (CASP) for qualitative studies checklist. However, most studies performed poorly on the third criterion, “appropriate research design (*n* = 9),” and the sixth criterion, “consideration of researcher-participant relationship (*n* = 12) (Figure 2).”

**Table 1:** Summary of included studies.

Author, year, country	Aim	Participants	Methods	Main findings
Tubic, 2023, Sweden	To explore patients’ experience of participation in care and feelings of safe care.	20 patients from surgical wards (Upper gastrointestinal surgery, acute surgery, and colorectal cancer surgery).	A descriptive qualitative study with manifest content analysis.	Feelings of being safe arising from a perception of good quality care. Experience good quality care Being informed contributes to feeling safe
Groves, 2023, United States	To describe safety and quality concerns experienced by hospitalized patients and families and factors and outcomes surrounding decisions about voicing concerns.	19 discharged inpatients or family member (10 were patients from adult patient care units and nine were parents of patients on pediatric units).	A qualitative descriptive design with directed content analysis of semi-structured interviews.	Main safety concerns Staff competency or knowledge Communication and coordination Potential treatment errors Care environment Factors influencing feeling safe Healthcare team member characteristics Communication and coordination Safe care expectations
Asikainen, 2023, Finland	To explore forensic psychiatric inpatients’ perceptions of patient safety.	65 forensic psychiatric inpatients.	A qualitative research using thematic analysis.	Psychological safety Care culture Patient-related themes Physical safety Environment Patient-related themes
Schaaf, 2022, Netherlands	To explore experiences of the safety of hospital-admitted patients in learning departments where students and nurses provide care together.	13 patients admitted to a learning department in the University Medical Center.	A qualitative explorative study with thematic analysis.	Having accountable nurses Trust through autonomy and support Taking time to communicate A safe learning environment with backup Being unaware of being in a learning department
Occelli, 2022, France	To describe patients’ own perspectives on the safety of the surgical care they received.	85 adult patients admitted for hospitalization in two orthopedic and in two digestive surgery wards in four hospitals.	A qualitative study was conducted based on interviews.	The trust in the surgeon The preoperative consultation
Barrow, 2022, United Kingdom	To understand how hospital inpatients across three different specialties conceptualize patient safety and develop a conceptual model that reflects their perspectives.	24 inpatients across three clinical specialties (medicine for the older adult, elective surgery and maternity).	A qualitative semi-structured interview using constant comparative analysis and memo-writing.	Patients Reporting my concerns Taking responsibility for myself Keeping an eye on&checking my care Following advice, rules&regulations Staff Demonstrating qualities&skills Performing clinical tasks Who is interacting with me Friends, family, and caregivers Being my advocate Supporting me Organization Maintaining the environment Cleaning having protocols&plans in place for safety
Jang, 2022, Korea	To understand the patients’ experience of safety with hospitalization, and identify the themes that constitute the patients’ feeling of safety during hospitalization.	35 participants.	A qualitative study, comprising five focus group discussions.	Patients who want to take initiative in controlling his/her reception of information Direct or indirect experience by others or media Effort to access and understand the medical information
				Hope to take a proactive role in decision-making based on active communication Healthcare providers who make the patient feel safe Physician’s professionalism and positive clinical outcome Attitude to fulfill the patients’ desire to know Treatment of patients with the highest priority Respecting patients’ information and privacy Hospital’s unstinted and generous support An organized communication system Sufficient arrangement of employees Efficient working process for healthcare provider Management of hospital facility and medical devices Public sentiment about national healthcare and safety Distrust of governmental programs to evaluate the level of institutional quality Trust and preference to a big private hospital Insensitivity toward safety that is prevalent in our society
Cutler, 2021, Australia	To explore how the physical and social environment of acute mental health units influences consumers’ perception and experience of safety.	15 people from acute mental health units.	A qualitative descriptive study using thematic analysis.	A supportive environment was experienced when consumers had privacy, felt safe from other consumers, and had meaningful activities to participate in within the acute mental health unit. Privacy Other consumers Meaningful activities
Cutler, 2021, Australia	To explore how consumers’ personhood influences their perception and experience of safety in acute mental health units.	15 people from acute mental health units.	A qualitative descriptive study using thematic analysis.	When participants’ innate worth was affirmed in their interactions with staff, participants felt safe. Seen as an equal Being respected Able to make choices
Cutler, 2020, Australia	To explore how nurses influence the perceptions and experience of safety among consumers who have been admitted to an acute mental health unit.	15 people from acute mental health units.	A qualitative descriptive study using thematic analysis.	The way nurses engaged in acute mental health units had a profound impact on participants’ sense of safety. Availability: “It’s about nurses spending time with you” Being responsive: “They would listen if you had a concern” Caring: “Little acts of kindness.”
Ellegaar, 2020, Denmark	To develop a grounded theory of the patients’ experiences with patient-controlled admission.	26 patients from 10 different mental health units.	A grounded theory.	Feeling safe Reversing the downward spiral Being self-determining Achieving calmness Receiving care Feeling unsafe Feeling overlooked Feeling uncertain
Piri, 2019, Sweden	To enhance our understanding of feelings of being safe or unsafe in psychiatric inpatient care.	17 adult patients from four settings: one general psychiatric, one psychiatric addiction, and two forensic psychiatric clinics.	A interview with open-ended questions using thematic content analysis.	Predictable and supportive services are necessary for feeling safe An unpredictable treatment process A need for structure and routines A desire for a friendly ward climate Communication and taking responsibility enhance safety A desire for communicative staff members Asking and waiting Taking responsibility Powerlessness and unpleasant encounters undermine safety Keeping away Powerlessness in relation to staff Unpleasant or violent co-patients
Lovink, 2015, Netherlands	To explore the experiences of safety of adult patients during their hemodialysis treatment.	12 hemodialysis patients received their outpatient treatment in a hemodialysis unit in a hospital.	A descriptive exploratory qualitative study using content analysis.	Insecurity Trust in the nurse Presence of nurse Patients’ need to control their situation
Stenhouse, 2013, United Kingdom	To understand the experience of being a patient on an acute psychiatric inpatient ward.	13 patients with a variety of diagnoses were recruited from an acute ward.	An unstructured interviewing using a holistic analysis.	Help Safety Power
Lasiter, 2011, United States	To report older adults’ perceptions of feeling safe in an intensive care unit.	10 older adults who were admitted to intensive care units.	A grounded theory study.	Proximity Oversight Predictability Initiative
Vaismoradi, 2011, Iran	To explore patients’ understandings and feelings of safety during hospitalization.	19 patients hospitalized in medical and surgical wards of a teaching hospital.	A qualitative design using a thematic analysis approach.	From attention to recovery Becoming hopeful of life Every event that disappointed patients or reduced their hope of recovery Not to be forgotten Maintaining life routines

### 3.2 Qualitative meta-synthesis

**Figure 3:**
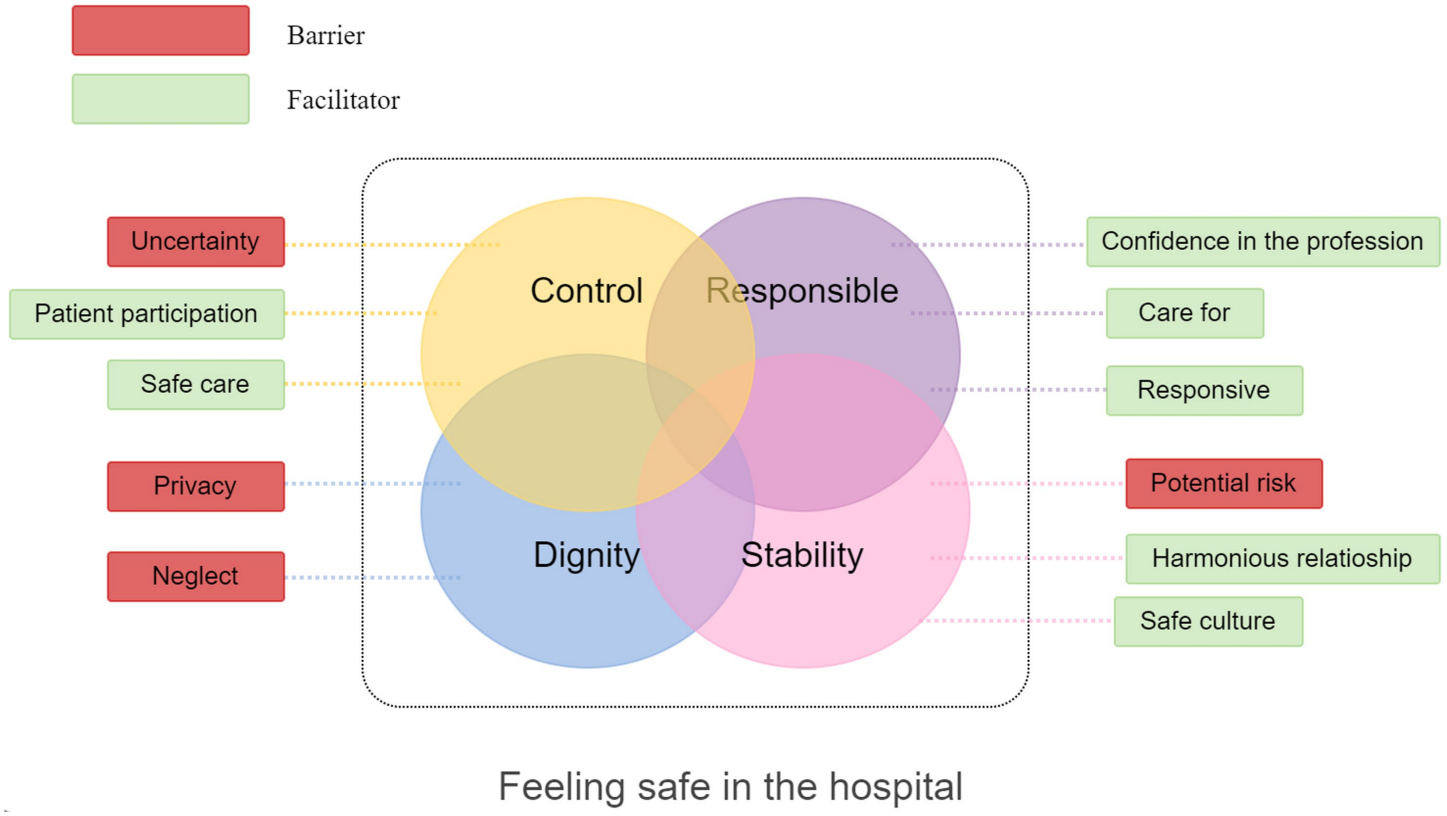
Feeling safe in the hospital.

We extracted 48 themes and 56 sub-themes from 16 studies. Next, four analytic themes relating to views on feeling safe were identified: control, responsible, dignity, and stability. These were underpinned by 11 descriptive themes. Across the four analytic themes, control included a barrier (Uncertainty) and two facilitators (Patient participation and safe care); responsible included three facilitators (Confidence in the profession, care for, and responsive); dignity included two barriers (Privacy and Neglect); stability included a barrier (Potential risk) and two facilitators (Harmonious and safe culture). A model of barriers and facilitators to feeling safe for inpatients involving the logical connection between these themes and sub-themes was constructed (Figure 3). Participant’s quotes taken directly from their original texts were used below to clarify these themes.

#### 3.2.1 Control

This theme focused on the patient’s sense of control over safety during hospitalization, including one barrier (Uncertainty) and two facilitators (Patient participation and safe care). Control can be explained that patients want to understand medical information, participate in meaningful care activities, and expect healthcare professionals to provide safe care service. However, patients felt uncertain due to a lack of medical knowledge and treatment information. Participants were concerned about not receiving the right medication, examination, and treatment (20). The uncertainty about the treatment weakened their perception and experience of safety and was a barrier to feeling safe for inpatients. Patients hoped to acquire information about their illnesses through all channels (6).

“*When I meet my healthcare professionals and talk about my illness, I repeatedly ask my physicians to confirm what I know and understand* (6).”

Some patients perceived many potential treatment risks (e.g., venipuncture pain, arteriovenous fistula) that made them feel unsafe and contributed to uncertainty, and they wanted to monitor and participate in their treatment (21). Patients felt safe when they actively participated in the medical process and made decisions based on physician advice and their own judgment (6). Besides, patients could prepare well for safety-related issues when they obtain adequate information about their treatment from healthcare professionals (6). Allowing patients to have control and make decisions for their treatment was another way of empowering them. Being able to make decisions gave patients a sense of safety since they had some control over their circumstances (10, 22).

“*What does safe care mean? It means being able to control and choose what happens to you* (10).” “*Being able to make choices and say concerns. We’re patients not prisoners* (9).”

In addition to participating in their treatment activities, patients also wanted to participate in activities they liked (9). These activities may include walking, watching TV, and living alone (9). Patients believed these meaningful activities could enhance their perception and experience of safety and contribute to safer care during hospitalization (9, 23). The third sub-theme of control was safe care. Patients expect to enjoy safe care service. Many participants expressed trust in the healthcare professionals, which provided an expectation of safe care (11). A previous pleasant hospitalization experience or familiarity with the hospital atmosphere also was a basis for their expectation of safe care (11).

“*I trusted my physician. I had an interview with him before all this happened. He seemed like he was a competent doctor* (11).”

#### 3.2.2 Responsible

This theme focused on providing patients with a sense of security from the perspective of healthcare professionals, including three facilitators (Confidence in the profession, care for, and responsive). Responsible meant medical staff possessed good professional qualities, cared for patients, and were promptly present on the patient’s side when they needed help.

Patients felt safe with the care they received due to feelings that medical staff were competent and performed their professional jobs well, which showed how patients’ feelings of safety were closely related to having confidence in healthcare professionals (23). Moreover, patients’ observations about the skills and knowledge of medical staff shaped their safe feelings (7). Patients felt safe when they believed the staff could provide routine treatment courses and deal with emergent issues (24). We described these characteristics as confidence in the profession, including knowledge, clinical skills, critical thinking, the ability to recognize problems, quick reaction, and thirst for knowledge.

“*You buy into that person, you put all your faith into their ability to finish their tasks* (7).” “*They cared for me, they knew what they were doing* (23).”

Participants reported a lack of communication with nurses made them feel unsafe (11, 25). They described that misunderstanding often happened to communication since nurses babbled and did not verify whether the patient understood the matter (25). From patients’ perspectives, ideal communication should involve being informed clearly and effective coordination between medical team members; examples were medical staff informing the patient and their caregivers about treatment information, being slow, being transparent, and providing physical and verbal reassurance (11).

“*I know that it’s hard for different teams to communicate, but doctors would just come in and they would hit you with message and there would not be a follow-up* (11).” “*They were communicative about what they were doing, why they were doing it. So, I never felt like I was in the dark, they kept the routines* (11).”

The second sub-theme of responsible was “care for,” including physical and psychological care. Participants reported an enhanced experience of safety when healthcare professionals expressed caring toward them (8). Healthcare professionals’ expressions of caring, conveyed by acts of empathy and kindness toward patients, made patients feel valued, and this made them feel safe (8). Another form of care was that the nurse was familiar with the patient’s treatment course. This familiarity was not only routine checks, but nurses should be attentive to the patient’s feeling of psychological safety. Patients reported that special attention, good communication, and a harmonious relationship with medical staff were essential to feel emotionally safe (21).

“*I could talk a lot about the acts and expressions of kindness that have made me feel safe* (8).” “*That gives a lot of confidence at that moment, you are not alone; there is someone with you who holds you* (21).”

For many participants, feeling safe meant healthcare professionals were present in the wards and stayed with patients (8, 21, 26). When staff was not in the wards, patients described feeling alone and without supervision, and their perception of safety was weakened (8). Moreover, it was necessary to be promptly responsive (22). Patients felt physically safe when staff was in their proximity, or staff quickly came into the wards when they called or pressed a button on an alarm system (21). In a word, presence meant that healthcare professionals spent time with patients, were responsive to their calls and familiar with them and their needs.

“*It’s not about giving someone medicines and giving them a meal*…*it’s about nurses spending time with you* (8).” “*You press a button already helps because you know someone is coming, making me calmer. Then it gets better* (22).”

#### 3.2.3 Dignity

This theme focused on patients’ dignity, including two barriers (Privacy and neglect). Dignity can be explained that medical staff created a safe privacy environment for patients, respected their privacy and preferences, and valued their needs. However, some participants described they could not control who could enter personal spaces (e.g., bathrooms and wards), which impaired their feeling of safety (9). Although most patients were accustomed to medical staff freely entering their individual spaces in this unique hospital environment, they expressed concerns about privacy (9). Furthermore, a mixed-gender ward also made patients unsafe, and the inability to have privacy was a barrier for inpatients to feel safe.

“*The doors could still be opened from the outside, and anyone could enter my bedroom when I am asleep* (9).”

Another aspect of dignified care was that patients hoped to be respected. When participants were asked to describe their sense of safety, they described it as caring for patients’ dignity and well-being (27). They hoped not to be neglected by healthcare professionals (27). Patients who felt neglected often stayed in their spaces and kept their concerns to themselves rather than communicating with someone (26). Therefore, being neglected by healthcare professionals was an important barrier to feeling safe for inpatients. Furthermore, patients expected staff to become familiar with their daily routines and respect their preferences (27). Patients felt safe when their dignity was protected, their preferences were heard, and they were treated courteously (10).

“*They are listening and asking question, and you feel that you are seen and heard. That’s probably the most important thing* (23).” “*The nurse came in my room and turned the TV off. She told me that I should sleep, but I wanted to watch TV. She should respect my choices* (27).”

#### 3.2.4 Stability

This theme focused on the internal and external hospital environment for safety, including one barrier (Potential risk) and two facilitators (Harmonious relationship and safe culture). Stability can be explained that the hospital had a stable environment, with harmonious relationships among medical team members and patients and a safe culture atmosphere.

In specific departments of hospitals (e.g., psychiatric departments), participants felt particularly vulnerable when sharing a ward with other patients (28). The potential risk was that some co-patients were perceived as scary or uncontrollable, and these patients had a tendency toward violence (20). Patients in the psychiatric wards were susceptible to the external environment, and lacking knowledge of their fellow patients made them feel unsafe (28). Participants expected the hospital to be a safe space and protect them safe from others (28).

“*I was in a dorm. If you are in a dorm, it’s very difficult because you do not know the state of mind of other patients* (28).” “*He checks that the coast is clear and then talks about horrendous assaults on women all the time* (20).”

A supportive environment enhances patients’ perception of safety (9). Their perceptions of safety were evoked through the observations, interactions, and encounters in the hospital (7). Patients felt safe when they saw the hospital environment was being cared for, cleaned, and maintained (7). In addition, trust in medical staff was a prerequisite for perceptions of safety, which based on interactions with the staff and their communication styles (29). Patients hoped to effectively interact with healthcare professionals and maintain a harmonious and trusted relationship with them. Certainly, patients also needed the support of relatives (7). Patients reported that participating relatives contributed to an increased safety experience and having close caregivers present also reduced their sense of loneliness (23). Harmonious relationships, clean and stable hospital environment, and rules and regulations would be related to safety culture, which was a facilitator to feeling safe for patients (6, 25).

“As long as you trust them…you feel safe (29).” “Then friends have been here and my children have been here and so I feel nice. It feels like I’m included in a context like that…I would have felt very lonely otherwise (23).”

## 4 Discussion

This study systematically synthesized 16 qualitative articles to explore the safe feelings of patients in the hospital. Through coding, constant comparison, and analysis, these experiences were organized into four themes (Control, responsible, dignity, and stability). We identified four barriers and seven facilitators to feeling safe for inpatients across four themes and further organized these findings into a model to present a way of understanding safe feelings.

Our results highlight the importance of patient participation, accountable staff, hospital support, and interactions, which is the primary way to make patients feel safe. These results support existing works on feeling safe (4, 11). In a previous concept analysis of feeling safe in the hospital, four defining attributes were identified: trust, cared for, presence, and knowledge (4). Some of our results closely align with these defining attributes. “Trust” could be found in the expectation of safe care of the first theme, control. “Cared for” is related to the attentiveness emphasized here in the sub-themes, care for and neglect. “Presence” is included in the sub-theme, responsive. Finally, “knowledge” could be attributed to the sub-theme, confidence in the profession. This concept analysis research conducted the work of synthesizing qualitative studies around the concept of feeling safe and identified the core defining attributes of the concept. Clarifying the concept of feeling safe could guide the construction of patient-centered care models and improve medical staff awareness of patients’ safe experiences. Based on existing meta-synthesis and new studies, our study refined barriers and facilitators for improving patients’ perception of safety from the themes of control, responsible, dignity, and stability, which could help healthcare organizations formulate safe care systems and adjust resource structures and further foster positive health, patient participation and service satisfaction at a population level.

Our model presents four barriers to feeling safe for inpatients: uncertainty, privacy, neglect, and potential risk. These four descriptive themes are derived from the summary of patient safety experience. Our study found that many patients worried about their safety during hospitalization (7, 11). Patients were concerned about the competency of novice staff and whether they could receive the correct medication and treatment (11). However, patients dared not voice their concerns since they were still determining if the problem was a high priority, which impaired their sense of safety (7). Therefore, listening to patients’ concerns and valuing their needs is essential to increase their sense of security. These findings also support existing studies regarding the significance of open safety communication and a reliable response to patients and caregivers who express concerns (11). In addition, some patients reported how the inability to control who could enter their personal spaces negatively impacted their perception of safety (9). During the COVID-19 pandemic, the ward and hospital environment has been altered; patients are concerned about unclean rooms, crowded unit design and infection risks related to the novel coronavirus (11). Restricted staff recruitment due to COVID-19 and the hospital staff’s overwhelming workload contributed to patient concerns. A comfortable environment was a significant factor in patients’ safe experiences, and lack of security is inconsistent with patients’ needs. Accordingly, healthcare professionals should pay more attention to keeping the environment clean and respecting their preferences, which requires careful consideration of care delivery models and facility design.

Our model presents seven facilitators to feeling safe for inpatients, which shows that patients’ sense of security comes from multiple layers, including patients, medical staff, diagnosis and treatment environment, and hospital rules and regulations. Patients hoped that medical staff had solid professional knowledge and practical skills, could spend time with them, and know their individual needs and preferences (8, 23). In emergency cases, medical staff could be actively responsive and solve the problem promptly, which is also their expectation of safe care during hospitalization (11, 21). Furthermore, our study shows that patients’ perception of safety includes physical and psychological safety, similar to the existing research (25, 30). Physical safety was that patients received a normal treatment course and did not suffer from treatment complications (21). In a previous study, psychological safety was considered as a key factor of better health care, involving those interested in high-quality care, open communication, and harmonious teamwork (30). In our included studies, patients reported feeling emotionally safe when physical safety conditions were satisfied (21). Physical safety appeared to be a requisite for emotional safety, and healthcare professionals had a core role in bringing about two forms of safety.

The medical delivery model increasingly emphasizes patient-centered care, advocates patient participation, and builds a harmonious patient safety culture. Patient safety culture was described as the attitudes, values, beliefs, and behaviors of medical staff shared in ensuring patient safety, which was conducive to reducing the incidence of adverse outcomes and benefiting staff well-being (3). Patient safety culture created an environment of openness and trust, enabling the team to concentrate on high-quality care and making patients feel safe. Certainly, creating a good safety culture requires the common efforts of medical and administrative staff, as well as the collaborative support of all stakeholders.

Our study identifies barriers and facilitators by summarizing patient safety experiences, which interplay and can be transformed into each other. Integrating these results in a qualitative meta-synthesis promises to enhance their impact on clinical practice and health policy and help medical staff to identify and overcome the potential barriers to implementing interventions in safe care. Patients have a sense of control over the disease when medical staff are responsible in their work, take the initiative to care for patients, and respect patients’ privacy and preferences. On this basis, the hospital provides a stable and familiar environment for patients, and patients would have a long-term sense of safety. Both individuals and organizations are responsible for promoting safe care through risk management, proactive policies, emergency interventions, and the construction of patient safety culture.

## 5 Limitations

Some limitations of our study should be acknowledged and considered. First, we only analyzed published quotes rather than the full text in the original studies, and we only included English research, which might impair the pluralism of the data. Second, our integration focused on qualitative research, while some cross-sectional studies also included factors related to patient’s safe perceptions. These limitations may contribute to selection, publication, and cultural biases. In addition, the quality assessment results showed that most included studies lacked consideration of researcher-participant relationships in the data analysis section, which might influence the original data quality. However, we prioritized participant perspectives and experiences by analyzing quotes, not the authors’ themes or interpretations. We expect that future research could address researcher reflexivity to improve the rigor of studies.

## 6 Conclusion

This study identifies key barriers and facilitators to feeling safe for inpatients, indicating that the safe perception is related to physical, emotional, relational, situational, and cultural dimensions. Moreover, we construct a model to reflect that feeling safe is a complex sense and interacts with four themes: control, responsible, dignity, and stability. Surrounding four themes and related barriers and facilitators, we outline principles for creating a safe environment and present strategies for improving patients’ hospitalization experience and ensuring patient safety. We believe that developing a model (including barriers and facilitators) for feeling safe would provide a valuable description of its nature and dimensions, in turn informing health policies and clinical practice in safe care.

## Data availability statement

The data sets used or analyzed during the current study are available from the corresponding author upon reasonable request.

## Author contributions

LY: Conceptualization, Formal Analysis, Investigation, Methodology, Writing – original draft. LL: Formal Analysis, Investigation, Methodology, Writing – review & editing. FW: Formal Analysis, Investigation, Writing – review & editing. FZ: Formal Analysis, Investigation, Writing – review & editing. XH: Conceptualization, Writing – review & editing.
