# Supplementary material for: Barriers and facilitators to feeling safe for inpatients: a model based on a qualitative meta-synthesis
Source: Front Public Health. 2024 Feb 28;12:1308258. doi: 10.3389/fpubh.2024.1308258 (PMC10933108; doi:10.3389/fpubh.2024.1308258)
Supplement: Supplementary file 1 [file Data_Sheet_1.docx]

Supplemental 1 Search terms

| **#** | **Search terms** |
| --- | --- |
| 1 | hospital |
| 2 | hospitalization |
| 3 | inpatient |
| 4 | department |
| 5 | 1 or 2 or 3 or 4 |
| 6 | feeling safe |
| 7 | sense of safety |
| 8 | patient safety |
| 9 | 6 or 7 or 8 |
| 10 | 5 and 10 |
